# MIND4-17 protects retinal pigment epithelium cells and retinal ganglion cells from UV

**DOI:** 10.18632/oncotarget.21131

**Published:** 2017-09-21

**Authors:** Chaopeng Li, Kang Yan, Wenqi Wang, Qing Bai, Changming Dai, Xiaofeng Li, Darui Huang

**Affiliations:** ^1^ Department of Ophthalmology, Huai’an First People's Hospital, Nanjing Medical University, Huai’an, China; ^2^ Department of Ultrasound, Huai’an First People's Hospital, Nanjing Medical University, Huai’an, China

**Keywords:** UV radiation, Nrf2, MIND4-17, retinal pigment epithelium cells, retinal ganglion cells

## Abstract

Nrf2 activation would efficiently protect retinal cells from UV radiation (UVR). Recent studies have developed a Nrf2-targeting thiazole-containing compound MIND4-17, which activates Nrf2 through blocking its association with Keap1. In the current study, we demonstrated that pretreatment with MIND4-17 efficiently protected retinal pigment epithelium (RPE) cells (RPEs) and retinal ganglion cells (RGCs) from UVR. UVR-induced apoptosis in the retinal cells was also largely attenuated by MIND4-17 pretreatment. MIND4-17 presumably separated Nrf2 from Keap1, allowing its stabilization and accumulation in retinal cells, which then translocated to cell nuclei and promoted transcription of ARE-dependent anti-oxidant genes, including *HO1*, *NQO1* and *GCLM*. Significantly, shRNA-mediated knockdown of Nrf2 almost completely abolished MIND4-17-induced cytoprotection against UVR. Further studies showed that MIND4-17 largely ameliorated UVR-induced ROS production, lipid peroxidation and DNA damages in RPEs and RGCs. Together, MIND4-17 protects retinal cells from UVR by activating Nrf2 signaling.

## INTRODUCTION

Retinal degenerative diseases are characterized by progressive loss of retinal cells [1–3]. UV radiation (UVR) and subsequent oxidative stresses are known as the major cause of damages to the resident retinal cells, including the retinal pigment epithelium (RPE) cells (RPEs) and retinal ganglion cells (RGCs) [4, 5]. Intense recent studies have been focusing on exploring novel strategies to protect retinal cells from UVR/oxidative stresses [6–9].

NF-E2-related factor 2 (Nrf2) is one extensively studied anti-oxidant signaling [10–13]. As a transcription factor, activated Nrf2 binds to the antioxidant responsive elements (AREs). It thus dictates transcription and expression of multiple genes, including those of endogenous antioxidants, phase II detoxification enzymes, along with other cellular defensive proteins [10–13]. Several key Nrf2-dependent genes have been identified, including *NAD(P)H quinone oxidoreductase 1 (NQO1)*, *heme oxygenase 1 (HO-1)*, γ-glutamylcysteine synthetase modifier subunit (GCLM) and *catalytic subunit (GCLC)*, among others [10–13]. Studies have demonstrated that Nrf2-dependent antioxidant response is a pivotal protection system against oxidative insults in mammalian cells [7, 14–16].

Nrf2 activation is tightly controlled by its inhibitor protein, Keap1 (Kelch-like ECH-associated protein 1) [13, 17, 18]. Nrf2's binding to Keap1 in the cytoplasm will dictate it to Cullin-3-dependent ubiquitination and proteasomal degradation. Following activation, Nrf2 will disassociate with Keap1 ubiquitin E3 ligase complex, causing it stabilization and accumulation [13, 17, 18]. Activated/stabilized Nrf2 translocates to cell nuclei and then binds to ARE, leading to multiple gene transcription.

Recent studies have developed a Nrf2-targeting thiazole-containing compound, namely MIND4-17 [19, 20]. This small molecule compound covalently modified a critical stress-sensor cysteine (C151) of Keap1, leading to Nrf2's departure from the ubiquitin E3 ligase complex. This would lead to Nrf2 stabilization, accumulation and nuclear translocation [19, 20]. Thus, treatment of this compound would induce profound Nrf2 activation. In the current study, we demonstrate that MIND4-17 efficiently protects RPEs and RGCs from UVR.

## RESULTS

### MIND4-17 protects retinal pigment epithelium (RPE) cells (RPEs) and retinal ganglion cells (RGCs) from UVR

**Figure 1:**
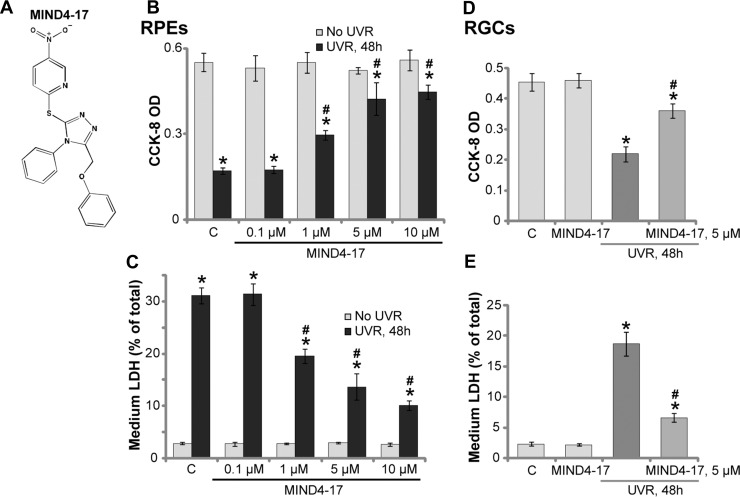
MIND4-17 protects retinal pigment epithelium (RPE) cells (RPEs) and retinal ganglion cells (RGCs) from UVR The molecule structure of MIND4-17 was presented (**A**). ARPE-19 cells (**B–C**) or primary cultured human RGCs (**D** and **E**) were pretreated for 30 min with applied concentration of MIND4-17, cells were then subjected to UV radiation (UVR, UVA2 + B, 30 mJ/cm^2^) and were further cultured for 48 hours; Cell viability was tested by the CCK-8 assay (B and D); Cell death was examined by LDH release in the conditional medium (C and E). For each assay, *n* = 5. “C” stands for untreated control cells. *p < 0.05 *vs.* “C” cells. ^#^*p* < 0.05 *vs.* “UVR” only cells. Experiments in this figure were repeated four times to insure consistency of results.

The molecule structure of MIND4-17 [19, 20] was presented in Figure 1A. We aim to study the potential effect of this Nrf2-inducing compound on UV radiation (UVR)-induced retinal cell damages. ARPE-19 cells are well-established human retinal pigmentation epithelial (RPE) cells [7]. Cultured ARPE-19 cells were treated with UVR (UVA2 + B, 30 mJ/cm^2^) [7, 21]. After 48 hours of culture, the Cell Counting Kit-8 (CCK-8) assay results in Figure 1B demonstrated that the viability of ARPE-19 cells was dramatically reduced following UVR, and the CCK-8 optic density (OD) decreased over 60% (Figure 1B). Significantly, pretreatment with MIND4-17 (at 1–10 μM) largely inhibited UVR-induced viability reduction in ARPE-19 cells (Figure 1B). MIND4-17-indued cytoprotection was concentration-dependent (Figure 1B). MIND4-17 at a low concentration (0.1 μM) was in-effective against UVR (Figure 1B).

Lactate dehydrogenase (LDH) release is often tested as a marker of cell death. Following UVR, the LDH level in the conditional medium of ARPE-19 cells was significantly increased (Figure 1C), indicating cell death. Pretreatment with MIND4-17 at 1–10 μM largely attenuated UVR-induced ARPE-19 cell death (LDH release, Figure 1C). Retinal ganglion cells (RGCs) are also main UVR-targeting cells in the retina [21, 22]. Here, we demonstrated that UVR similarly induced viability loss (CCK-8 OD reduction, Figure 1D) and cell death (LDH release, Figure 1E) in primary human RGCs [21, 22]. Importantly, such effects by UVR were largely attenuated with pretreatment of MIND4-17 (5 μM) (Figure 1D and 1E). It should be noted that treatment with MIND4-17 alone at tested concentration failed to change viability and death of the retinal cells (Figure 1B–1E). Together, these results demonstrate that MIND4-17 protects human RPEs and RGCs from UVR.

### MIND4-17 inhibits UVR-induced apoptosis in RPEs and RGCs

**Figure 2:**
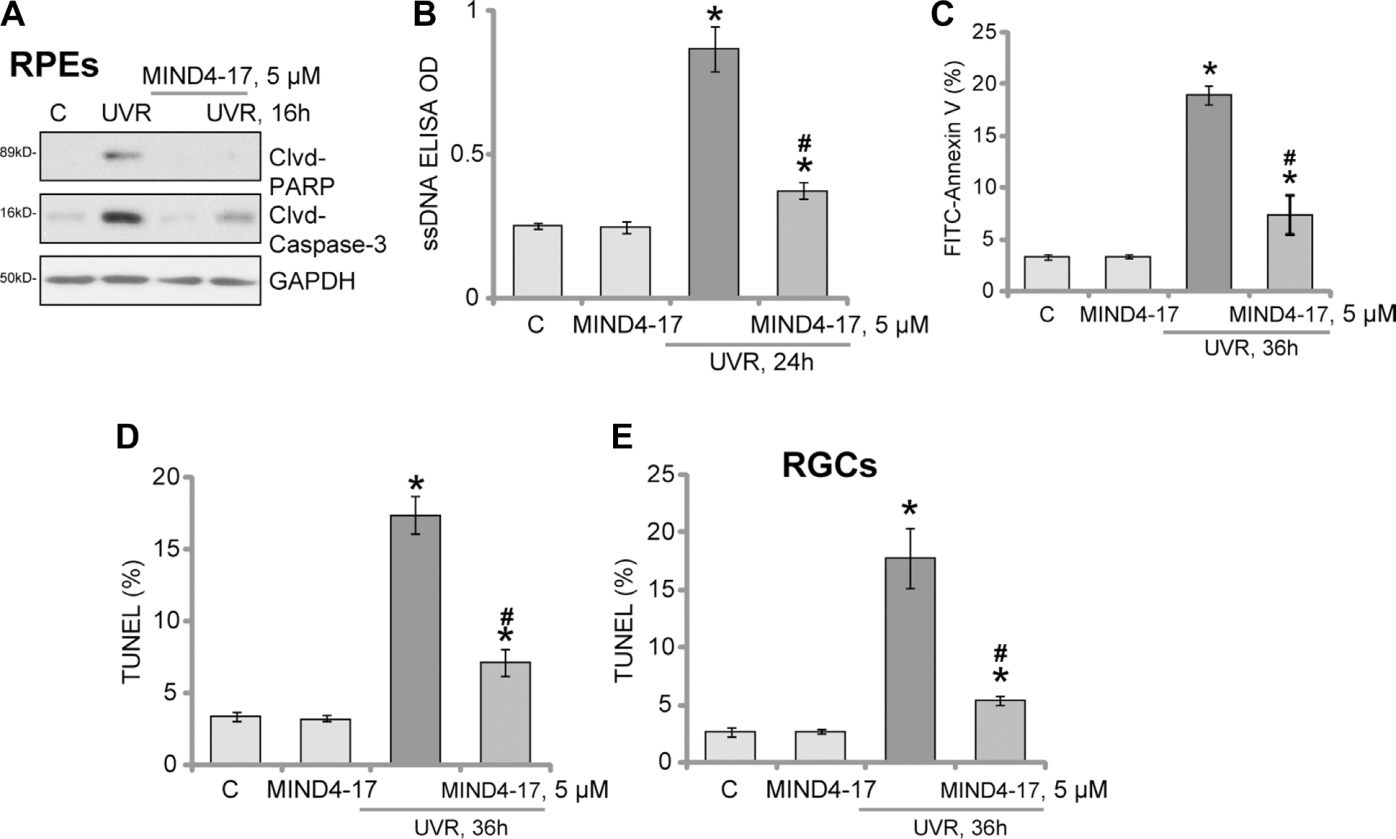
MIND4-17 inhibits UVR-induced apoptosis in RPEs and RGCs ARPE-19 cells (**A–D**) or primary cultured human RGCs (**E**) were pretreated for 30 min with MIND4-17 (5 μM), cells were then subjected to UV radiation (UVR, UVA2 + B, 30 mJ/cm^2^) and were further cultured for applied time; Expressions of cleaved-PARP (“Clvd-PARP”) and cleaved-caspase-3 (“Clvd-Caspase-3”) were tested (A, GAPDH was shown as the loading control); Cell apoptosis was tested by the assays mentioned in the text (**B–E**). Annexin V ratio included both early (PI negative) and late (PI positive) apoptotic cells (**C**). For TUNEL assay, at least 200 cells in five random views (1×100 magnification) of each condition were analyzed to calculate TUNEL ratio (D and E). “C” stands for untreated control cells. *p < 0.05 *vs.* “C” cells. ^#^*p* < 0.05 *vs.* “UVR” only cells. Experiments in this figure were repeated three times to insure consistency of results.

The potential effect of MIND4-17 on UVR-induced retinal cell apoptosis was also tested. As shown in Figure 2A, in the ARPE-19 cells, 16 hours after UVR (UVA2 + B, 30 mJ/cm^2^), expressions of both cleaved-caspase-3 and cleaved-PARP [poly (ADP-ribosyl) transferase] were both increased (Figure 2A). Meanwhile, UVR-induced significant production of single strand DNA (ssDNA), which is the characteristic marker of cell apoptosis (Figure 2B). Such effects by UVR were largely inhibited with pretreatment of MIND4-17 (5 μM) in ARPE-19 cells (Figure 2A and 2B). These results suggest that MIND4-17 possibly inhibits UVR-induced RPE cell apoptosis. Indeed, further studies displayed that MIND4-17 (5 μM) pretreatment efficiently inhibited UVR-induced increase of Annexin V-positive (Figure 2C) and TUNEL-positive (Figure 2D) ARPE-19 cells. The similar results were also obtained in the primary human RGCs, where MIND4-17 (5 μM, 30 min pretreatment) inhibited UVR-induced apoptosis induction (TUNEL cell increase, Figure 2E). MIND4-17 alone was in-effective to cell apoptosis in the tested retinal cells (Figure 2A–2E). Together, we demonstrate that MIND4-17 inhibits UVR-induced apoptosis in human RPEs and RGCs.

### MIND4-17 activates Nrf2 signaling in retinal cells

**Figure 3:**
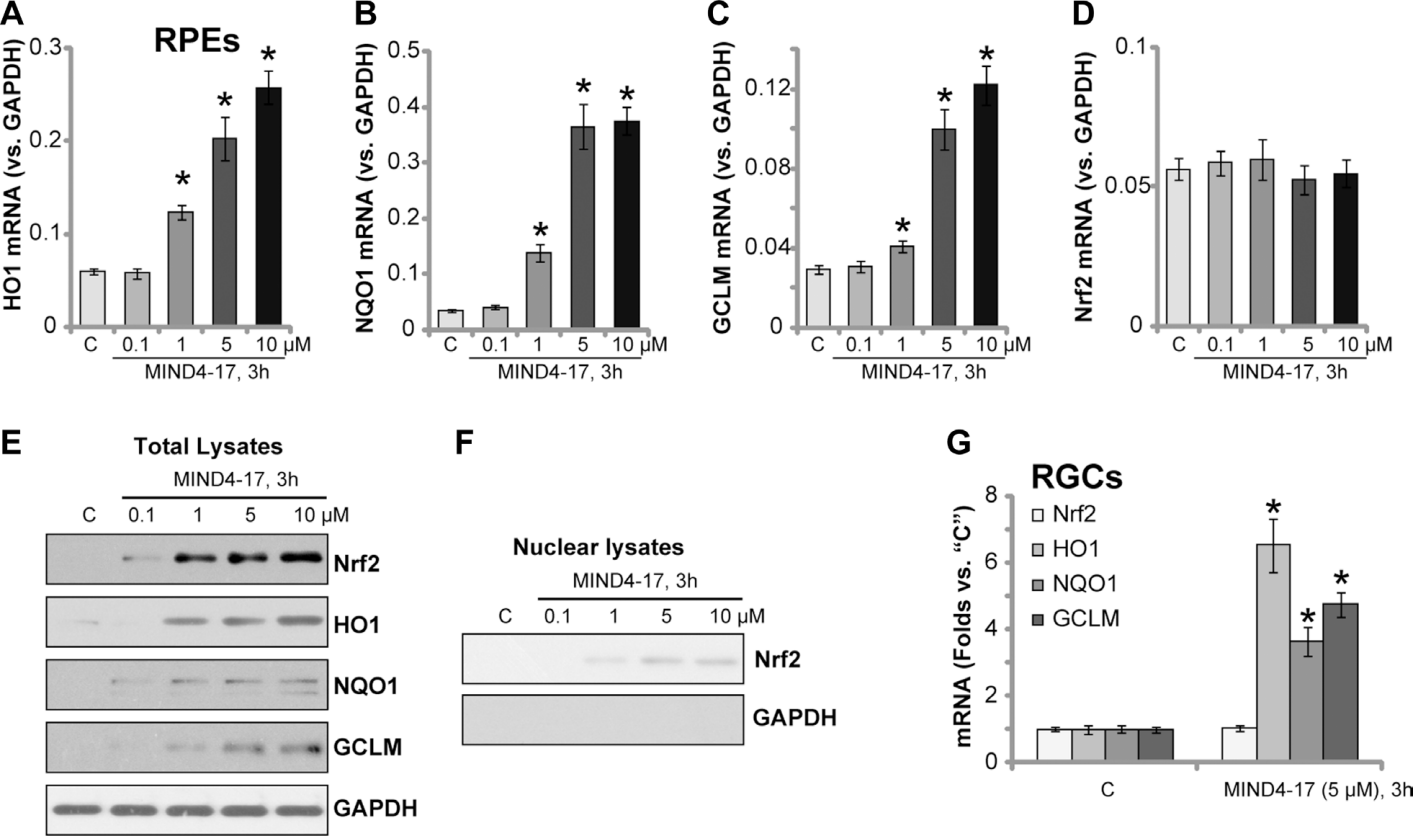
MIND4-17 activates Nrf2 signaling in retinal cells ARPE-19 cells (**A–F**) or primary cultured human RGCs (**G**) were treated with applied concentration of MIND4-17, cells were further cultured for 3 hours; The real-time quantitative PCR (RT-qPCR) assay was employed to test mRNA expressions of listed genes (A–D and (G), *GAPDH mRNA* was tested as the internal control); Listed proteins in total cell lysates (E) and nuclear fraction lysates (F) were also tested by Western blotting assay (GAPDH was tested as the loading control, which was absent in the nuclear fractions). “C” stands for untreated control cells. *p < 0.05 *vs.* “C” cells. Experiments in this figure were repeated three times to insure consistency of results.

Activation of Nrf2 signaling pathway can inhibit UVR-induced damages in retinal cells [6, 7, 23, 24]. MIND4-17 is a Nrf2-inducing compound [19, 20]. We therefore tested Nrf2 signaling in MIND4-17-treated retinal cells. The real-time quantitative PCR (“RT-qPCR”) assay results displayed that treatment with MIND4-17 at 1–10 μM significantly increased mRNA expressions of several Nrf2-dependent genes [14, 15, 25], including *heme oxygenase-1* (*HO-1*, Figure 3A), *NAD(P)H quinone oxidoreductase 1* (*NQO1*, Figure 3B) and γ-glutamyl cystine ligase modulatory subunit (*GCLM*, Figure 3C). The effect by MIND4-17 was dose-dependent (Figure 3A–3C). On the other hand, *Nrf2 mRNA* level was unchanged before and after t MIND4-17 treatment (Figure 3D). Nrf2 protein level was yet significantly increased in MIND4-17-treated RPE cells, suggesting Nrf2 stabilization (Figure 3E). Protein expressions of HO1, NQO1 and GCLM were also boosted following MIND4-17 (1–10 μM) treatment (Figure 3E). Importantly, we found that stabilized Nrf2 translocated to cell nuclei after treatment with MIND4-17, and the nuclear Nrf2 protein level was significantly increased (Figure 3F). Based on these results, we propose that MIND4-17 treatment possibly separates Nrf2 from Keap1, thus allowing stabilization and accumulation of Nrf2, which then translocates to cell nuclei, causing transcription of ARE-dependent genes, *i.e.* *HO1*, *NQO1* and *GCLM*. In the primary human RGCs, MIND4-17 (5 μM, 3 hours) treatment also increased mRNA expressions of *HO1*, *NQO1* and *GCLM*, but not Nrf2 (Figure 3G). Therefore, MIND4-17 apparently activates Nrf2 signaling in retinal cells.

### Nrf2 is required for MIND4-17-mediated retinal cytoprotection against UVR

**Figure 4:**
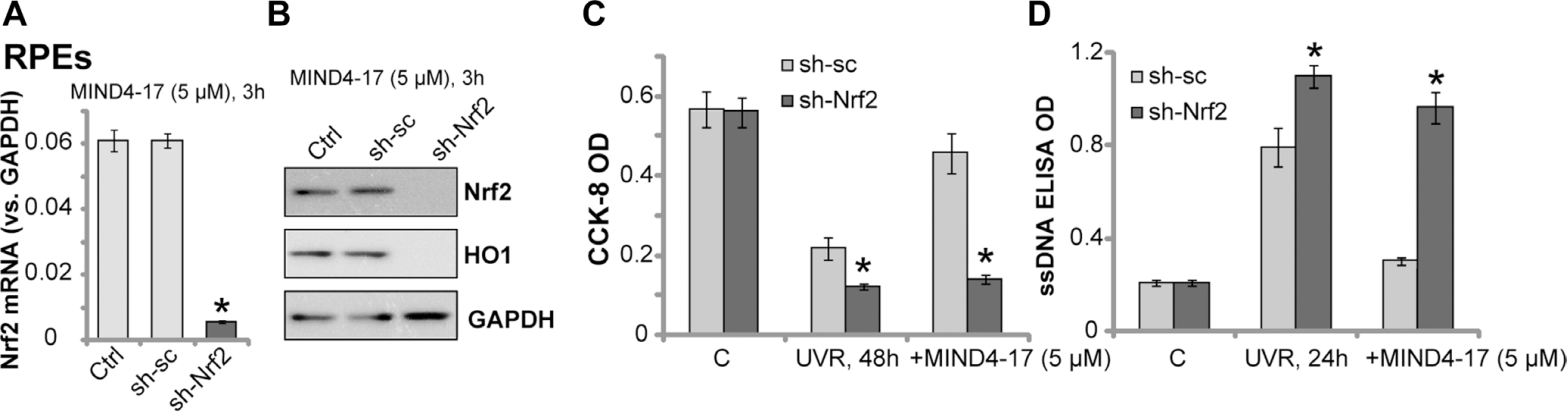
Nrf2 is required for MIND4-17-mediated retinal cytoprotection against UVR Stable ARPE-19 cells, expressing scramble control shRNA (“sh-sc”) or Nrf2 shRNA (“sh-Nrf2”), were treated with MIND4-17 (5 μM) for 3 hours, *Nrf2 mRNA* (**A**) and listed proteins (**B**) were tested by RT-qPCR assay (*GAPDH mRNA* was tested as the internal control) and Western blotting assay (GAPDH was tested as the loading control), respectively; Cells were exposed UV radiation (UVR, UVA2 + B, 30 mJ/cm^2^) and cultured for applied time; Cell survival and apoptosis were tested by CCK-8 assay (**C**) and ssDNA ELISA assay (**D**), respectively. “Ctrl” stands for parental ARPE-19 cells (A and B). For each assay, *n* = 5. *p < 0.05 *vs.* “sh-sc” cells. Experiments in this figure were repeated three times to insure consistency of results.

In order to test that Nrf2 signaling activation is required for MIND4-17-mediated cytoprotection, short hairpin RNA (shRNA) method was employed to knockdown Nrf2 in retinal cells. As described [7, 24], the Nrf2 shRNA-containing lentiviral particles (purchased from Santa Cruz Biotech) were added to ARPE-19 cells. Stable cells were then selected by puromycin. The applied Nrf2 shRNA dramatically downregulated *Nrf2 mRNA* expression in the stable cells (Figure 4A). Since basal Nrf2 protein level was low in the ARPE-19 cells (see Figure 3), we stimulated cells with MIND4-17 (5 μM, 3 hours). Western blotting assay results confirmed that the applied Nrf2 shRNA also dramatically downregulated Nrf2 protein in MIND4-17-treated ARPE-19 cells (Figure 4B). Downstream HO1 expression was also largely inhibited (Figure 4B). Remarkably, UVR-induced cell death (CCK-8 OD reduction, Figure 4C) and apoptosis (ssDNA ELISA OD increase, Figure 4D) were intensified in Nrf2-shRNA-expressing ARPE-19 cells. These results suggest that Nrf2 activation might also be important for the protection against UVR, which is consistent with previously findings [6, 7]. Significantly, MIND4-17-mediated cytoprotection against UVR was almost completely abolished in Nrf2-silenced cells (Figure 4C and 4D). MIND4-17 was also most ineffective against UVR in Nrf2-shRNA expressing cells (Figure 4C and 4D). The similar results were also obtained in the primary human RGCs (Data not shown). These results suggest that Nrf2 is required for MIND4-17-mediated retinal cytoprotection against UVR.

### MIND4-17 efficiently attenuates UVR-induced oxidative stress in retinal cells

**Figure 5:**
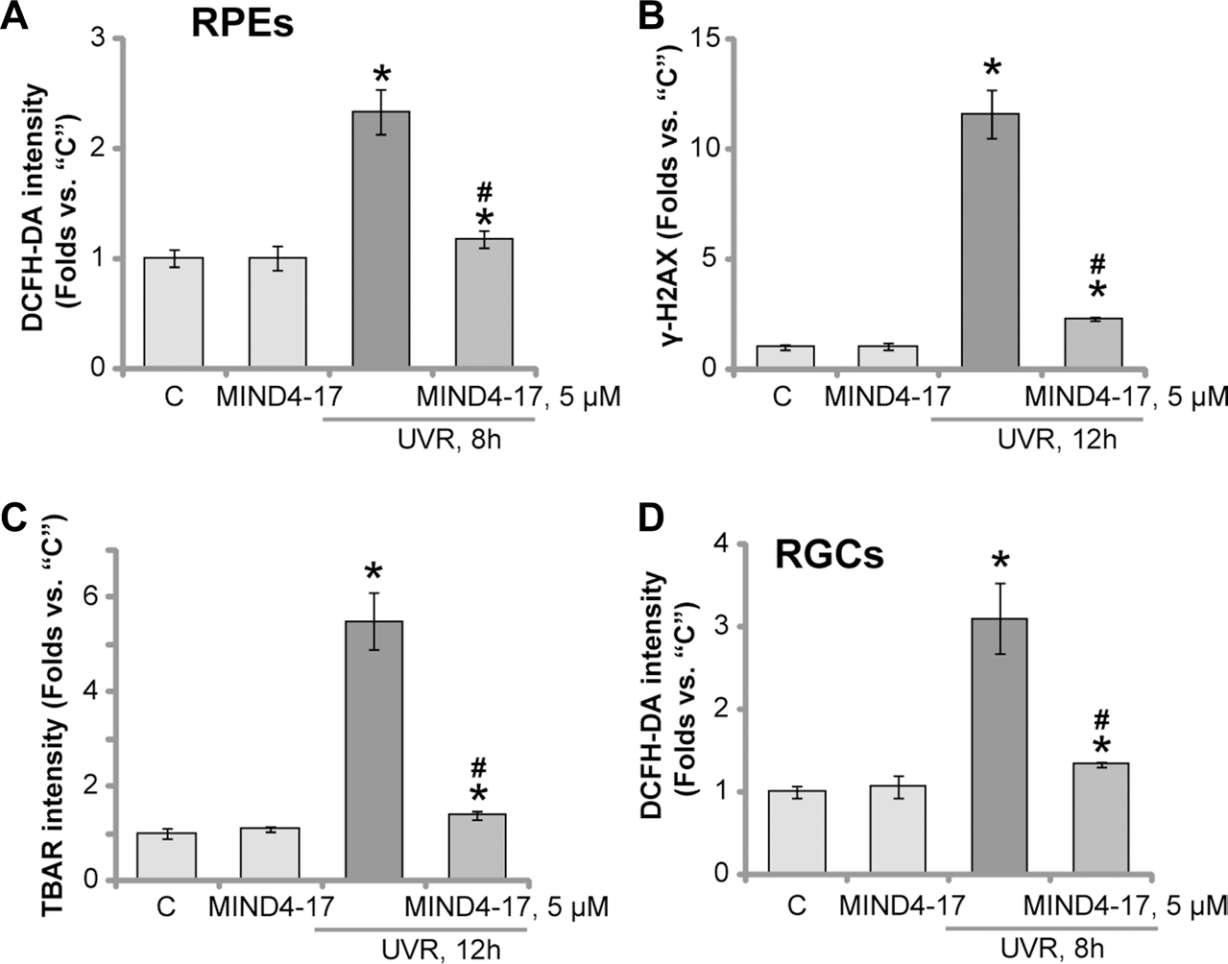
MIND4-17 efficiently attenuates UVR-induced oxidative stress in retinal cells ARPE-19 cells (**A–C**) or primary cultured human RGCs (**D**) were pretreated for 30 min with MIND4-17 (5 μM), cells were then subjected to UV radiation (UVR, UVA2 + B, 30 mJ/cm^2^) and were further cultured for applied time; ROS production was tested by DCFH-DA intensity assay (A and D); DNA damages and lipid peroxidation were tested by γ-H2AX assay (B) and TBAR activity assay (C), respectively. “C” stands for untreated control cells. *p < 0.05 *vs.* “C” cells. ^#^*p* < 0.05 *vs.* “UVR” only cells. Experiments in this figure were repeated three times to insure consistency of results.

Reactive oxygen species (ROS) production and following oxidative stress are major causes of cell death by UVR [26, 27]. Nrf2 signaling is a well-established anti-oxidant signaling [10, 12, 17, 28]. In line with previous findings [6, 7, 29, 30], UVR radiation to ARPE-19 cells induced significant ROS production, which was tested by increase of DCFH-DA intensity (Figure 5A). UVR-induced ROS production was followed by increase of DNA damages (γ-H2AX intensity increase, Figure 5B) and lipid peroxidation (TBAR intensity increase, Figure 5C). Remarkably, such effects by UVR were largely attenuated by pretreatment of MIND4-17 (5 μM) (Figure 5A–5C). In the primary human RGCs, MIND4-17 (5 μM) pretreatment also inhibited ROS production by UVR (Figure 5D). These results demonstrate that MIND4-17 efficiently attenuates UVR-induced oxidative stress in retinal cells.

## DISCUSSION

In the pathology of AMD and other retinal degenerative diseases, UVR and subsequent oxidative stresses cause direct damages to resident retinal cells, *i.e.* RPEs and RGCs [6–8, 21, 31, 32]. Existing recent studies have demonstrated that activation of Nrf2 could be a fine strategy to protect retinal cells from UVR and/or oxidative stress [6–8, 21, 31, 32]. For instance, Zhang *et al.,* showed that Salvianolic acid A protected RPEs from oxidative stress through activation of Nrf2-HO1 signaling [8]. Similarly, the other Traditional Chinese Medicine, 3H-1, 2-dithiole-3-thione (D3T), activated Nrf2 signaling to protect RPEs from UVR [6]. SC79, a novel AKT activator [7, 33, 34], protected RPEs from UVR through activating AKT downstream Nrf2 signaling [7]. Additionally, microRNA-mediated silence of Keap1 also activated Nrf2 signaling in RPEs and RGCs, therefore offering protection against UVR-induced oxidative stress [21].

Recent studies have characterized MIND4-17 as a unique and highly selective small molecule activator of Nrf2 [19, 20]. MIND4-17-induced Nrf2 activation is initially through covalent modification of the Keap1 sensor-cysteine C151 [19, 20]. This modification mimics the effects of oxidative stress, which causes Keap1 conformational change and arrests of the Keap1-Nrf2 complex [19, 20]. This would release Nrf2 from the from the ubiquitin E3 ligase complex, causing its stabilization and accumulation in the cytoplasm. It is followed by nuclear translocation of Nrf2 and de novo synthesis of ARE-response genes [19, 20]. Unlike other known Nrf2 activators, this small molecule compound directly and uniquely disassociates the Nrf2-Keap1 complex. It therefore activates Nrf2 signaling at an extremely high efficiency [19, 20].

In the present study, we show that MIND4-17 treatment presumably separated Nrf2 from Keap1, thus allowing its stabilization and accumulation in retinal cells. Stabilized Nrf2 by MIND4-17 translocated to cell nuclei, causing transcription and expression of multiple ARE-dependent anti-oxidant genes, including *HO1*, *NQO1* and *GCLM*. Subsequently, activation of Nrf2 by MIND4-17 largely ameliorated UVR-induced ROS production, lipid peroxidation and DNA damages in RPEs and RGCs. Retinal cell death and apoptosis by UVR were also dramatically alleviated by MIND4-17. Importantly, we demonstrate that Nrf2 is required for MIND4-17's actions in retinal cells. shRNA-mediated knockdown of Nrf2 almost completely reversed MIND4-17-induced retinal cytoprotection against UVR.

## MATERIALS AND METHODS

### Reagents, chemicals and antibodies

Based on the structure in previous studies [19, 20] (also see Figure 1A), MIND4-17 was synthesized and verified by Minde Biotech (Suzhou, China). Puromycin was obtained from Sigma-Aldrich (Nanjing, China). The reagents for cell culture were provided by Gibco (Guangzhou, China). Antibodies against Nrf2, HO1, NQO1, GCLM and GAPDH were purchased from Santa Cruz Biotech (Beijing, China). Antibodies for cleaved-caspase-3 and cleaved-PARP were provided by Cell Signaling Tech (Nanjing, China).

### Culture of RPEs

The established ARPE-19 cells were provided as a gift from Dr. Jiang [6, 35, 36]. The DMEM plus fetal bovine serum (FBS) medium was employed for ARPE-19 cell culture.

### Culture of primary human RGCs

The primary-cultured human RGCs were also provided by Dr. Jiang [21]. The primary human cells were maintained as described previously [21]. Primary human RGCs at passage 3–8 were utilized for further experiments.

### UV radiation

UV radiation (UVR, UVA2 + B, 30 mJ/cm^2^) to the cultured RPEs and RGCs was described previously [6, 7, 29, 30]. After UVR, cells were maintained in the conditional medium, and cultured for described time.

### Cell viability and cell death assays

Testing of cell viability by the Cell Counting Kit-8 (CCK-8, Dojindo Laboratories, Kumamoto, Japan) [37] as well as cell death assay by lactate dehydrogenase (LDH) release assay (Takara, Tokyo, Japan) were extensively described in early studies [38]. CCK-8 optic density at 450 nm was recorded. LDH content in the conditional medium was normalized to total LDH [38, 39].

### ssDNA ELISA assay of cell apoptosis

DNA formamide denaturation and subsequent the production of single strand DNA (ssDNA) is characteristic marker of cell apoptosis, which was tested by the ApoStrandTM ELISA apoptosis detection kit (BIOMOL International, Plymouth Meeting, PA). ELISA OD at 450 nm was recorded.

### Assessment of apoptosis by Annexin V FACS assay

Following the applied treatment, retinal cells were harvested, washed and resuspended in binding buffer with 1 μL of Annexin V-FITC and 1 μL of propidium iodide (PI) (Biyuntian, Wuxi, China). After incubation for 20 min at room temperature, cells were then tested by flow cytometry via CellQuest software (BD Biosciences, Shanghai, China). Annexin V ratio was recorded [8, 39].

### TUNEL assay

After the described treatment, retinal cells were further stained with TUNEL and DAPI florescence dyes (Sigma, Shanghai, China). TUNEL ratio (vs. DAPI staining) was calculated [40].

### Western blotting assay

Cells were lysed via the described lysis buffer [41]. Determination of the protein concentration was though the Bio-Rad Protein Assay (Bio-Rad, Shanghai, China). Protein concentration (μg/mL) was measured at 690 nm. The detailed protocol for Western blotting assay and data quantification were described previously [42, 43]. Cell nuclei were isolated by the “cell nuclei isolation kit” (Sigma) [8].

### Real-time quantitative PCR

Total RNA was extracted by TRIzol reagents (Invitrogen, Shanghai, China) and then reversely transcribed using a PrimeScript RTreagent kit (Takara Biotechnology, Japan). Real-time quantitative PCR (“RT-qPCR”) assay was performed by a SYBR Premix Ex TaqTM kit on the ABI-7700 fast PCR system (Takara Biotechnology, Japan). mRNA primers for human *HO-1, NQO1 and GLCM* and *GAPDH* were described previously [44]. *GAPDH* *mRNA* was tested as the reference gene. The 2^–ΔΔCt method was employed to^ calculate relative expression of targeted *mRNA*s.

### Nrf2 shRNA

The Nrf2 shRNA lentiviral particles (sc-37030-V, Santa Cruz Biotech, Shanghai, China) and the scramble control shRNA lentiviral particles (sc-108080, Santa Cruz Biotech) were both commercial available. The shRNA lentivirus was added to ARPE-19 cells for 12 hours. Afterwards, puromycin (2.5 μg/mL) was added to select stable cells for 5–6 passages. Nrf2 knockdown was verified by both Western blotting assay and RT-qPCR assay.

### Reactive oxygen species (ROS) assay

ROS intensity in the control and UVR-treated retinal cells was measured by the carboxy-H2DCF-DA dye (Invitrogen, Shanghai, China) assay [45]. Following the treatment, cells were incubated with 1 μM of carboxy-H2-DCFDA for 120 min under the dark, which were then washed with warm PBS and immediately tested by a spectrofluorometer at excitation and emission wavelengths of 485 and 535 nm, respectively. The results were expressed as increase in fluorescence with respect to control cells.

### Lipid peroxidation assay

Thiobarbituric acid reactive substances (TBAR) level was examined to reflect the production of toxic aldehyde resulting from oxidative fatty acyl degradation, the malondialdehyde (MDA). The detailed protocol was described previously [45, 46].

γ-H2AX assay of DNA damages. ARPE-19 cells were fixed in ice-cold ethanol, which were then incubated with a mouse monoclonal anti-γ-H2AX antibody (Santa Cruz Biotech). Afterwards, the FITC-conjugated anti-mouse secondary antibody (Santa Cruz) was then added. Cells were then subjected to FACS assay to determine the γ-H2AX percentage, reflecting DNA damage intensity [47].

### Statistics

The results were presented as the mean ± standard deviation (SD). One-way ANOVA was employed to examine the significant differences between groups using SPSS 17.0 (SPSS, Chicago, CA). Values of *p* < 0.05 were considered statistically significant.

## CONCLUSIONS

Together, we conclude that targeting Nrf2 by MIND4-17 efficiently protects RPEs and RGCs from UVR.
